# Prediction of the Toxicity of Binary Mixtures by QSAR Approach Using the Hypothetical Descriptors

**DOI:** 10.3390/ijms19113423

**Published:** 2018-10-31

**Authors:** Ting Wang, Lili Tang, Feng Luan, M. Natália D. S. Cordeiro

**Affiliations:** 1 College of Chemistry and Chemical Engineering, Yantai University, Yantai 264005, China; 18865557652@163.com (T.W.); tangtang20160216@sina.com (L.T.); 2 LAQV/REQUIMTE, Department of Chemistry and Biochemistry, Faculty of Sciences, University of Porto, 4169-007 Porto, Portugal; ncordeir@fc.up.pt

**Keywords:** mixture, toxicity, multiple linear regression (MLR), radial basis function neural networks (RBFNNs)

## Abstract

Organic compounds are often exposed to the environment, and have an adverse effect on the environment and human health in the form of mixtures, rather than as single chemicals. In this paper, we try to establish reliable and developed classical quantitative structure–activity relationship (QSAR) models to evaluate the toxicity of 99 binary mixtures. The derived QSAR models were built by forward stepwise multiple linear regression (MLR) and nonlinear radial basis function neural networks (RBFNNs) using the hypothetical descriptors, respectively. The statistical parameters of the MLR model provided were N (number of compounds in training set) = 79, R^2^ (the correlation coefficient between the predicted and observed activities)= 0.869, LOOq^2^ (leave-one-out correlation coefficient) = 0.864, F (Fisher’s test) = 165.494, and RMS (root mean square) = 0.599 for the training set, and N_ext_ (number of compounds in external test set) = 20, R^2^ = 0.853, $q_{\mathrm{ext}}^{2}$ (leave-one-out correlation coefficient for test set)= 0.825, F = 30.861, and RMS = 0.691 for the external test set. The RBFNN model gave the statistical results, namely N = 79, R^2^ = 0.925, LOOq^2^ = 0.924, F = 950.686, RMS = 0.447 for the training set, and N_ext_ = 20, R^2^ = 0.896, $q_{\mathrm{ext}}^{2}$ = 0.890, F = 155.424, RMS = 0.547 for the external test set. Both of the MLR and RBFNN models were evaluated by some statistical parameters and methods. The results confirm that the built models are acceptable, and can be used to predict the toxicity of the binary mixtures.

## 1. Introduction

It has been widely accepted that the environmental pollutants usually exist and play a role in the form of mixtures, rather than as single chemicals. Thus, the mixture toxicity has attracted more attention of scientists in the past several decades [1,2,3]. Up to now, researchers have proposed two different approaches to evaluate the toxicity of mixtures, that is, the experimental toxicity tests to experimentally measure the toxicity of a whole mixture [4,5,6], and computational toxicology methods, such as quantitative structure–activity relationship (QSAR) studies, to predict the toxicity of the mixture [7,8,9,10]. For the experimental method, in the process of the determination of the toxicity of mixtures, individual compounds and combinations of arbitrary mixtures need to be tested one by one, and the combination of various compounds is infinite in the process of production. Furthermore, the last step of the experiment is usually to use animals as targets, which is difficult to achieve and ethically complicated. However, QSAR, as a computational method, has been used for almost six decades, and was widely applied in physical chemistry, pharmaceutical chemistry, environmental chemistry, toxicology, and other research fields [10]. It has been proven that it can be used to evaluate the properties, activities, and toxicities as effectively as the alternative methods to the experimental methods.

As for the recent QSAR methodology for the assessment of mixture toxicity, a QSAR model was formulated by using the similarity parameter (λ) to predict of the toxicity of equitoxic and nonequitoxic mixtures. From the results of this QSAR model, it can be seen that the joint effects of a mixture were determined by the concentration ratios of the individual components [11]. The protein receptor interaction energy (E_binding_) was used to establish a model for the evaluation of the differences between chronic and acute mixture toxicity [12]. The CORAL software was used as a tool to model the toxicity of 50 binary mixtures of halogenated benzene for *Photobacterium phosphoreum*. The authors came to the conclusion that the half maximal effective concentration (pEC_50_) increases when there are chlorine, bromine, and oxygen atoms in the molecule structure, however, the pEC_50_ decreases if there is nitrogen atom [13]. In the study of mixture toxicity, the molecular docking study between nonpolar narcotic chemicals (halogenobenzene, alcohols, and perfluorinated carboxylic acids (PFCAs)) and lipid membranes was performed, to obtain the binding energy (E_binding_). E_binding_ was then used to build a QSAR model. The authors declared that the new calculated method of the octanol/water partition coefficient of chemical mixtures (K_owmix_) by E_binding_ was proposed [1]. Later, the liposome–water partition coefficient (K_lipw_) was also used to build a QSAR model for 64 polar and nonpolar baseline toxicants measured by the bioluminescence inhibition in *Vibrio fischeri*. Then, the predicted values were used to evaluate the effect-based water quality trigger value (EBT-EC_50_) of mixture chemicals [14]. Yao et al. developed a QSAR model for the prediction of mixture toxicities by two parameters, E_binding_ and logK_ow(mix)_. In this study, the different mixtures (aldehydes and cyanogenic toxicants; triazines and urea herbicide; sulfonamides and trimethoprim toxicants) binding to different sites on different (or same) target proteins were considered [15]. We also performed the QSAR studies on a group of nonpolar narcotic compounds, including 9 PFCAs, 12 alcohols, and 8 chlorobenzenes and bromobenzenes by linear MLR and nonlinear RBFNN method. The predictive values are in good agreement with the experimental ones [10]. Based on the mixture toxicity mechanism, the formula between mixture toxicity and hormetic effect was built, and it can be used to predict the mixture toxicity from effect level at the low concentration. The authors also declared that the method can be further used for the prediction of mixture toxicity at any effect level from individual toxicity [16]. A general baseline toxicity QSAR model was performed also by using liposome–water distribution ratios as descriptors to assess the cytotoxicity of nonpolar, polar, and ionizable chemicals, and their mixtures, in the bioluminescence inhibition assay with *Aliivibrio fischeri* [3].

From the above research, the computational methods of mixture toxicity assessment have lasted and been developed over decades. The toxicity of mixtures has usually been evaluated by two methods, CA (concentration addition) and IA (independent action). It should be noted that, in these studies, the descriptors used to predict the toxicity are mostly the more effective two, E_binding_ and the octanol/water partition coefficient (logk_ow_). In the present work, we try to find other classical or defined hypothetical descriptors to relate the structure characteristic and the mixture toxicity, instead of the normally used E_binding_ and logk_ow_. In addition, the possible relationships between the single chemicals or their mixtures, and the molecular descriptor, are considered.

## 2. Results and Discussion

### 2.1. MLR Results

According to the method of calculation of the molecular descriptors, a total of 614 descriptors which encode kind of significant features of molecules were obtained. Following the heuristic method (HM) of the descriptor selection, the 132 descriptors were left after removing the descriptors that did not obey the four rules. Finally, the descriptor dataset was refined to 106 for the MLR analysis by removing the highly relevant descriptors.

Through the forward regression method, a model of three descriptors (max partial charge for a C atom (Zefirov’s PC (partial charge)) ($Q_{\mathrm{Max}}^{C}$), average complementary information content (order 2) (ACIC2), number of triple bonds (NTB) was finally obtained for the pure chemicals in this study. The equation is as follows:

(1) $$-\log\left({\mathrm{EC}}_{50}\right)=0.236-0.769\mathrm{NTB}+1.609\mathrm{ACIC}2+55.778Q_{\mathrm{Max}}^{C}$$

N = 55, R^2^ = 0.736, LOOq^2^ = 0.720, F = 47.334, RMS = 1.042 (2)

The above equation is for single pure compounds only. The predicted −log(EC_50_) values are shown in Table 1 as well as the name, Chemical Abstracts Service (CAS) number, and the experimental −log(EC_50_) values of the single chemicals.

The statistical results showed an acceptable correlation between the three descriptors and the toxicity of these single compounds. However, in terms of the toxicity of the mixture, whether the above descriptors or their combinations have any correlation with the binary mixture toxicity needs to be studied. It has been shown and confirmed that for the docking effect of any type of theses mixtures, their descriptors are shown to be only simple addition [15]. Therefore, the total effect of the mixture is quantified with the descriptors previously selected, expressed as the formula below:

(3) $$D=\sum x_{i}D_{i}$$

where *D_i_* represents the selected descriptor of the single pure compounds, and *x_i_* represents the ratio of toxic unit. D, the hypothetical descriptor, is only a numerical operator that sums each value of the selected descriptor in the mixture, considering its contributions.

In this study, for each binary mixture, three hypothetical descriptors were formed by $Q_{\mathrm{Max}}^{C}$, ACIC2, NTB and the fractional concentrations of the mixture. That is, the hypothetical descriptor is the sum of each descriptor of the pure compound, multiplied by the fraction of it in the mixture. Then, using the hypothetical descriptors, the 79 combinations belonging to the training set were used to build the model. The selected descriptors and their chemical meaning, along with the statistical parameters, were listed in Table 2. At the same time, the intercorrelation of the descriptors was evaluated when the model was built, as shown in Table 3. Pair correlations among the variables are far below 0.80 [17], indicating that each descriptor can be considered independent, which prevent grave over-fit of chance correlation effects happening. The corresponding variation inflation factors (VIF) and mean effect (MF) values of the three descriptors used in the model were also given in Table 2. From Table 2, we can see that the VIF value of each descriptor is less than 2, which is below the critical point 10 as an indicator of severe or serious multicollinearity [18]. The MF value of each descriptor is listed in this table. The value and the sign of them indicates the relative importance of a descriptor [19]. The MF values of descriptors $Q_{\mathrm{Max}}^{C}$, ACIC2, and NTB decrease in turn, indicating that their contribution to the model is also reduced.

**Figure 1:**
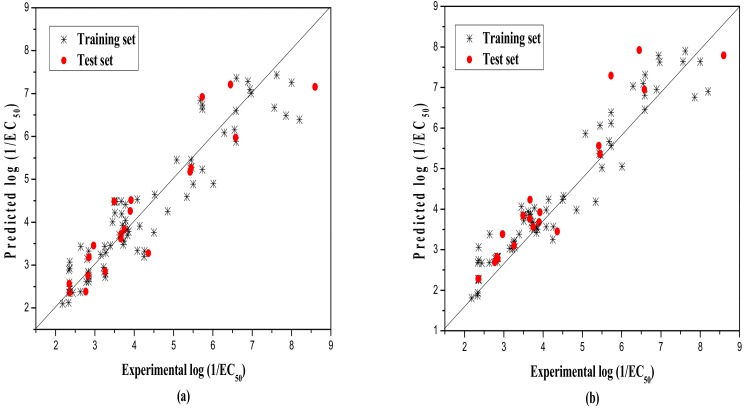
Plot of the predicted versus experimental −log(EC_50_) including the training and the test set by MLR model (**a**) and by RBFNN model (**b**).

**Figure 2:**
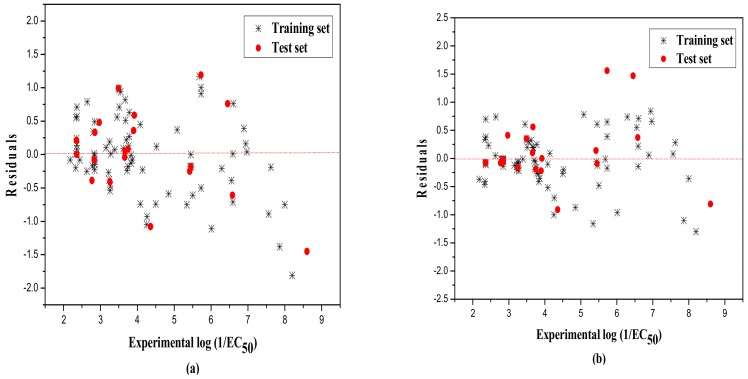
Residuals of the training and test set by MLR (**a**) and RBFNN (**b**).

Lastly, the model was evaluated by the external test set, whereby the statistic values are N_ext_ = 20, R^2^ = 0.853, $q_{\mathrm{ext}}^{2}$ = 0.825, F = 30.861, and RMS = 0.691. Our model also meets more stringent requirements, as shown in Table 4. That is, $q^{2}=0.825>0.5,R^{2}>0.6,R^{2}-R_{0}^{2}=0.004<0.3,k=0.983\approx 1,$ giving very good results. $R_{0}^{2}$ is the coefficient of determination (predicted vs observed activities), $R_{0}^{\text{'}2}$ is the coefficients of determination (observed vs. predicted activities through the origin), and k and k’, are the corresponding slopes of the regression lines through the origin. The predicted versus experimental −log(EC_50_) values of MLR model for the training and test sets are shown in Table 5, and plotted in Figure 1a. Moreover, Figure 2a shows the scatter plot of residuals of the whole dataset.

### 2.2. Model Applicability Domain Analysis and Improved MLR Model

**Figure 3:**
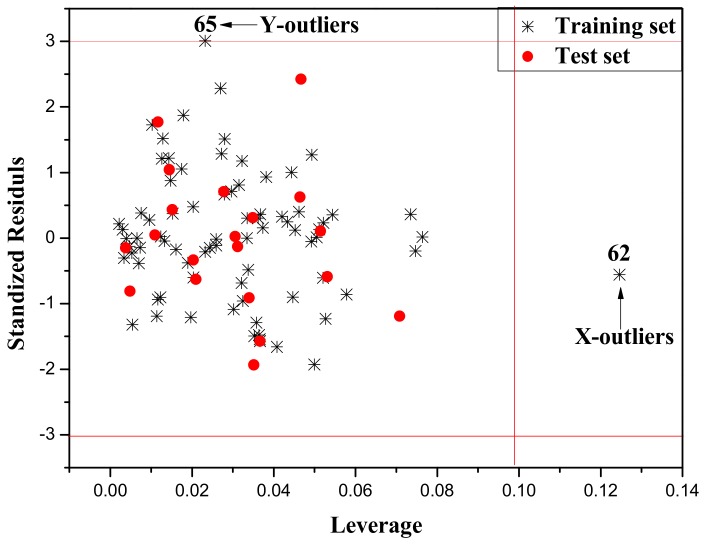
The William plot of the training and external test set.

The Williams plot (Figure 3) was used to visualize the application domains of the built model. In this plot, the horizontal (standardized cross-validated residuals) and vertical (leverage) straight lines represent the normal control values of Y-outliers and X-outliers, respectively. The limit of X-coordinate is set as *3m/n*, where *m* is the number of descriptors in the model, and *n* is the number of compounds belongs to training set. The normal control values for Y-outliers (standardized cross-validated residuals) was set as ±3σ. From Figure 3, there is a X-outlier (combination 62) and a Y-outlier (combination 65) in this model, and all of them belong to the training set.

From the molecular structures of the two compounds in the outlier mixture, we can clearly see that they both have chlorine atoms (Cl) in combination 62, while, for combination 65, there are Cl and sulfur atom (S) atoms, both as single compounds. While the combinations, except 62 and 65, do not have this structure feature, it could be speculated that negatively charged atoms can have an abnormal effect on −log(EC_50_) values.

After eliminating the two outliers in the model, an improved model was obtained using the left compound with the same descriptors. The relationship is $-\log\left({\mathrm{EC}}_{50}\right)=-0.397-0.619\mathrm{NTB}+1.740\mathrm{ACIC}2+64.914Q_{\mathrm{Max}}^{C}$. The statistical parameters are N = 77, R^2^ = 0.872, LOOq^2^ = 0.866, F = 165.068, and RMS = 0.566. From the results, one can see that it is a little better than the original one.

### 2.3. RBFNN Results

In general, nonlinear studies considering the nonlinear relationship between descriptors and activity usually show better results than linear ones in QSAR studies. In this paper, the nonlinear RBFNN method was employed to further verify this. The inputs of RBFNN were the same hypothetical descriptor (D) used in the MLR model, and the outputs are the same target −log(EC_50_) values. The same as with the MLR studies, the training set was used to establish the model, and the test set was used to evaluate the model. In the model building process, a three-layer net with 2-n_k_-1 was designed, where 2, n_k_, and 1 represent the number of the units in the input, the hidden, and the output layer, respectively. The width (r) of the RBF function was optimized by calculating its range, from 0.1 to 4, with the increment of 0.1. Finally, a 2-14-1 RBFNN model was built with the width of 1.2 and hidden neurons of 14. The statistical analysis results are, for training set, N = 79, R^2^ = 0.925, LOOq^2^ = 0.924, F = 950.686, and RMS = 0.447 and, for the test set, N_ext_ = 20, R^2^ = 0.896, $q_{\mathrm{ext}}^{2}$ = 0.890, F = 155.424, and RMS = 0.547. The predicted values of the final optimized nonlinear models, as well as their corresponding residuals, are also shown in Table 5. The predicted values vs the experimental values and the distribution of the residuals for the whole dataset are displayed in Figure 1b and Figure 2b. To further assess the predictive power of the model established by the RBFNN method, parameters, such as $\frac{\left(R^{2}-R_{0}^{2}\right)}{R^{2}},k,k^{,}$, etc. are also calculated as in the MLR model, and the results are shown in Table 4.

In all, it can be seen from the results that both the linear MLR and nonlinear RBFNN model can predict the toxicity of the mixture satisfactorily. The linear model is simple, intuitive, and easy to be used by the researcher, and the nonlinear model can give more accurate prediction results. The important thing is that we must know the three descriptors of each pure compound. In doing so, we can give the prediction of the toxicity of this group of compounds within the application domains.

### 2.4. Validation Results of the Models

In the present study, a Y-randomization test was developed 10 times for both of the linear and nonlinear models, and the R^2^ and LOOq^2^ values of the two models are shown in Table 6. For the MLR and RBFNN models, the average values of R^2^ were 0.0266, 0.0251, and the average values of LOOq^2^ were 0.0141, 0.0124, respectively. The above low average values for R^2^ and LOOq^2^ values of the models, which imply that both models are robust, and have no chance correlation or structural redundancy.

Further, a fivefold cross-validation algorithm (leave many out cross-validation (LMO)) was applied for validation of the stability of the two models. The members selected for each group (i.e., groups A, B, C, D and T) were shown in Table 5. In this table, for example, A+B+C+D means that the subsets A, B, C, D were selected as the training set, while the sunset T as test set. The R^2^, F, and RMS values for each validation, along with their average values, are shown in Table 7 (for the MLR model) and Table 8 (for the RBFNN model), respectively. As can be seen, both models are stable, judging from the obtained values for the average training quality (MLR model: R^2^ = 0.865, RMS = 0.607; RBFNN model: R^2^ = 0.917, RMS = 0.486) and for the average predicting quality (MLR model: R^2^ = 0.872, RMS = 0.643; RBFNN model R^2^ = 0.923, RMS = 0.468).

### 2.5. Interpretation of Model Descriptors

In the present study, three descriptors were selected for the model, namely $Q_{\mathrm{Max}}^{C}$, ACIC2, and NTB According to the computed MF value (see Table 2), $Q_{\mathrm{Max}}^{C}$ was the biggest one, so it plays the most important role in the process of model buildings. The positive sign indicated that the −logEC_50_ values increased with its increase, and vice versa. $Q_{\mathrm{Max}}^{C}$ is an electrostatic descriptor, which reflects how charge is distributed in the molecules partial surface area. $Q_{\mathrm{Max}}^{C}$ also affects the ability of the compound to be a H-bond acceptor. The stronger the electronegativity is, the easier it is to form hydrogen bonds, which leads to the enhancement of toxicity. Regarding the constitutional descriptor NTB

(the number of triple bonds), which mainly includes –CC (compound 31^#^) and –CN (compound 14^#^–24^#^) in this paper, it has a negative effect on the toxicity of the compounds. Average complementary information content (order 2) (ACIC2) belongs to topological descriptors. It describes the size, shape, and branching information of the molecules, and reflects the diversity of atomic and structural constitution of organic molecules. From the coefficient of the model, one can see that it has a positive effect on the toxicity of the compounds.

## 3. Materials and Methods

### 3.1. Datasets

In the present study, a group of 55 compounds, including 13 aldehydes (AHs) and 11 cyanogenic (CGs) as group 1, 10 triazines (TAs) and 10 urea (UE) herbicides as group 2, and 10 sulfonamides (SAs) and 1 trimethoprim (TMP) as group 3, were obtained from the literature by Yao et al. [15]. These compounds are widely present in the environment, and have an adverse effect on the environment and human health in the form of monomers or mixtures. The name, CAS number, and the experimental −log(EC_50_) values of the single chemicals were listed in Table 1, and the unit of EC_50_ is mol/L. The binary mixture toxicity of 99 combinations, along with the single chemicals in the mixtures, the ratio of toxic unit, as well as the experimental values of mixture (−log(EC_50mix_)), were listed in Table 5. The single compounds in Table 1 were used to select the proper descriptors, which were highly related to the toxicity of pure compounds. In doing so, the selected ones were then used to generate new descriptors for the building of the QSAR model of the mixture in Table 5. The dataset of the combinations in Table 5 were also randomly divided into a training set (including 79 combinations) to build the models, and a test set (including 20 combinations, marked with *) to evaluate the predictive power of models.

### 3.2. Molecular Descriptors Generation and Selection

To obtain the descriptors of the compounds, each structure of the single compounds was drawn in ISIS Draw 2.3 (MDL Information Systems, Inc., San Ramon, CA, USA) [20]. The preliminary molecular geometry optimization was done in HyperChem software (Hypercube, Inc., Waterloo, ON, Canada) [21] by the molecular mechanics MM^+^ force fields. Then, the further optimization of the molecular structures was carried out by semi-empirical PM3 method using the Polak–Ribière algorithm, until the root mean square gradient was 0.01 kcal/mol [22]. Finally, an optimization was also done in MOPAC software package (Indiana University, Bloomington, IN, USA) at the same root mean square gradient [23]. After that, the structure files exported from HyperChem and MOPAC were transferred into CODESSA software (University of Florida, Gainesville, FL, USA) [24] for the purpose of calculation of descriptors, which mainly include constitutional, topological, geometrical, electrostatic, and quantum-chemical descriptors. In addition, the logP descriptor, which cannot be calculated by CODESSA, was obtained by HyperChem and, then, was added to the descriptors pool [18].

After calculating the descriptors, the heuristic method (HM) was used to find the proper descriptors associated with the toxicity of single compounds; this was done by using the experimental −log(EC50) values of single chemicals as the end-point activity values. This method has been approved to be usefully used in the field of quantitative structure–activity relationship (QSAR) [10], quantitative structure–property relationship (QSPR) [25], and quantitative structure–toxicity relationship (QSTR) [26].

However, we all know that a considerable portion of these descriptors are constant and highly intercorrelated, requiring further screening. According to the CODESSA program, the choice of best descriptors by the heuristic method (HM) follows the following principles: descriptors must be obtained for each compounds; the magnitude between the descriptors should be large; the F-test’s value of the descriptor must be larger than 1.0 in the one-parameter correlation; and descriptors whose *t*-values are larger than the user-specified value (0.1) are more preferred. By doing so, the descriptors can be arranged in order of the influence of the one-parameter correlations.

Following the data reduction, forward stepwise multiple linear regression (MLR) method was used to select the suitable descriptors. In this process, the descriptor was added, one by one, until there was no further significant improvement in the statistics of the model. It should be noted that an increase of the “R^2^” value less than 0.02 was chosen as the breakpoint criterion.

### 3.3. Multiple Linear Regressions (MLR)

In the QSAR studies, the multiple linear regressions (MLR) method is often considered as a simple but powerful approach to regression problems when there are two or more than two independent variables. The goal is to find a mathematical function using the selected training set compounds which best describe the desired activity *Y* (here −log(EC_50_) values), as a linear combination of the *X*-variables (the molecular descriptors) with the regression coefficients *b_n_*. The equation is as follows:

(4) $$Y=b_{0}+b_{1}x_{1}+b_{2}x_{2}+\cdots +b_{n}x_{n}$$

Some statistical parameters, including R^2^ (the correlation coefficient between the predicted and observed activities), LOOq^2^ (leave-one-out correlation coefficient), RMS (root mean square error), F (Fisher’s statistics) [27], etc., are normally used to evaluate the stability of the model. For the purpose of estimation of the predictive ability of the model, some other statistical characteristics should also be considered: $R_{0}^{2}$ (the coefficient of determination, predicted vs observed activities), $R_{0}^{\prime 2}$ (the coefficients of determination, observed vs predicted activities through the origin), and k and k’, are the corresponding slopes of the regression lines through the origin [28].

(5) $$q^{2}>0.5$$

(6) $$R^{2}>0.6$$

(7) $$\frac{\left(R^{2}-R_{0}^{2}\right)}{R^{2}}<0.1\mathrm{or}\frac{\left(R^{2}-R_{0}^{,2}\right)}{R^{2}}<0.1$$

(8) 0.85≤k≤1.15 or 0.85≤k′ ≤1.15

Finally, Y-scrambling techniques should be used to rule out the possibility of chance correlation, and to inspect for reliability and robustness by permutation testing. Also, multicollinearity between the selected descriptors can be detected by calculating their variation inflation factors (VIF), as follows [29]:

(9) $$\mathrm{VIF}=\frac{1}{1-r^{2}}$$

In this equation, *r* represents the correlation coefficient between each pair of the selected descriptors. In order to evaluate the relative importance and the contribution of each descriptor in the model, the value of the mean effect (MF) is calculated by the following equation [30]:

(10) $$MF_{j}=\frac{\beta_{j}\sum_{i=1}^{i=n}d_{ij}}{\sum_{j}^{m}\beta_{j}\sum_{i}^{n}d_{ij}}$$

where *MF_j_* is the mean effect for the considered descriptor *j*, *β_j_* is the coefficient of the descriptor *j* in the model, *d_ij_* is the value of the target descriptors for each molecule, and *m* and *n* is the number of descriptors and compounds being used to build the model.

The MF value indicates the relative importance of a descriptor compared with the other descriptors in the model, and its sign exhibits the variation direction in the values of the activities resulting from an increase (or a reduction) of this descriptor value.

### 3.4. Radial Basis Function Neural Networks (RBFNNs)

For MLR method, all works were achieved by means of building the best multivariate linear model between molecular descriptors and the −log(EC_50_) values. However, there are also many other approaches that were used in the analysis of nonlinear QSAR data, such as Radial basis function neural network (RBFNN), for considering the nonlinear behavior of the desired activity. The principles of it have been mentioned elsewhere [31,32]. The RBFNN is a standard feed forward neural network characterized by a set of inputs and a set of outputs. Between the inputs and outputs, there is a layer of processing *n* units called hidden units. There is no connection between the neurons in the given layer, but each neuron in each layer is well connected to the next layer. The input layer does not handle any input information, and its only role is in the distribution of input to the hidden layer. The output layer gives the results of the hidden layer with a linear transformation. It is important that each hidden layer unit plays the role by a nonlinear function, which can be used to deal with the input information from the previous layer.

The most common use of RBF is the Gaussian function characterized by the center (*c_j_*) and width (*r_j_*). The RBF function realizes the nonlinear transformation by measuring the Euclidean distance between the input vector (*x*) and the radial basis function center (*c_j_*):

(11) $$h_{j}=exp\left(-\|x-c_{j}{}^{2}\|/r_{j}^{2}\right)$$

where *h_j_* represents the output of the *j*th RBF unit, *c_j_* represents the radial basis function center, and *r_j_* is the width of the unit. The linear transformation of the output layer is defined as

(12) $$y_{k}\left(x\right)=\overset{n_{h}}{\underset{j=1}{\sum }}w_{kj}h_{j}\left(x\right)+b_{k}$$

where *y_k_* represents the *k*th output unit for the input vector *X*, *w_kj_* represents the weight connection between the *k*th output unit and the *j*th hidden layer unit, and *b_k_* is the respective bias.

In the performance of the development of the RBFNN model, the center (*c_j_*) and width (*r_j_*) should be determined. In our study, a forward subset selection routine was used with a constant of Gaussian functions for all the units. The width (*r_j_*) was adjusted by changing its range from 0.1 to 4, with the increment of 0.1. After doing that, the connection weight between the hidden and output layer was selected by a least-squares solution. In all the optimal process, root mean square (RMS) was used as the error function. This process is completed by MATLAB (Available online: https://www.mathworks.com/products/matlab/). Like the MLR method, RBFNN is also evaluated with parameters such as RMS, R^2^, and others.

### 3.5. Applicability Domain (AD) of the Model

For a useful model, the applicability domain (AD) of the model should be given. The applicability domain (AD) of a QSAR model is a theoretical region in the space defined by the compounds in the training set. It characterizes the nature of the chemicals that can be used in the built model. That is, AD defines a theoretical region which can be used to predict the new compounds, even if they have not been tested by the experimental method [33]. There are some methods to achieve this purpose. In this study, a Williams plot, i.e., a plot of standardized residuals (*R*) vs leverages was used [34]. The leverage, *h_i_*, can be calculated by the formula [33], as follows:

(13) $$h_{i}=x_{i}^{T}{\left(x^{T}x\right)}^{-1}x_{i\;}\left(i=1,\;2,\;\cdots ,\;n\right)$$

where *h_i_* characterizes the leverage of a compound, *x_i_* represents the descriptor row vector of the studied compound, and *x* represents the whole matrix of the descriptor values of compounds in the training set. T is matrix or vector-transposed symbol.

### 3.6. Validation of QSAR Models

#### 3.6.1. Y-Randomization Test

Y-randomization (also called Y-scrambling or response randomization) is considered to be the most powerful validation procedure to protect against the risk of chance correlation. In this test, the dependent-variable vector, Y-vector, is randomly shuffled, and a new QSAR model is established using the initial independent-variable matrix [28]. The process is repeated many times, if in each case, the scrambled data gives much lower R^2^ and LOOq^2^ values than the original data that can be used to build the model, then, a robust model was obtained [35].

#### 3.6.2. Leave Many Out Cross-Validation

In addition, we also use leave many out cross-validation (LMO) to further evaluate the robustness of the model. Unlike LOO, the internal test set for LMO is for a group of compounds instead of single one. It is generally believed that the obtained model is robust, if a QSAR model has a high average R^2^ in the LMO cross-validation.

## 4. Conclusions

Prediction of the toxicity of a sequence of mixture was accomplished by the MLR (multiple linear regression) and RBFNN (radial basis function neural network) methods. The hypothetical descriptors (D), derived from the normal $Q_{\mathrm{Max}}^{C}$, NTB and ACIC2 descriptors were successfully used to measure the contributions of the components of a mixture to the overall activity. The MLR is an acceptable and easy method and, with regard to the RBFNN model, it can predict the mixture toxicity more accurately. In addition, statistical results show that the developed descriptors are effective and feasible for evaluating the toxicity of mixture compounds. Thus, the present study shows the viability of applying QSAR tools to predict the binary toxicity. Furthermore, the results of this study provided useful insights on the characteristics of the structures that most affect the toxicity.

## Figures and Tables

**Table 1:** Toxicity data of the single chemicals.

No.	Single Chemicals	CAS	−logEC_50_ (mol/L)		Residual
			Experimental	Predicted	
1#	Acetaldehyde	75-07-0	2.36	3.177	0.817
2#	Propanal	123-38-6	2.72	3.212	0.492
3#	Butyraldehyde	123-72-8	3.25	3.224	−0.0265
4#	Valeraldehyde	110-62-3	3.27	3.628	0.358
5#	Benzaldehyde	100-52-7	3.43	4.552	1.122
6#	*p*-Nitrobenzaldehyde	555-16-8	4.28	3.634	−0.646
7#	*p*-Terephthaldehyde	623-27-8	4.07	4.880	0.810
8#	*p*-Chlorobenzaldehyde	104-88-1	3.97	3.876	−0.094
9#	*p*-Bromobenzaldehyde	1122-91-4	4.3	3.861	−0.437
10#	*p*-Hydroxybenzaldehyde	123-08-0	4.54	3.777	−0.763
11#	*p*-Methyl benzaldehyde	104-87-0	3.82	4.030	0.210
12#	*p*-Methoxybenzaldehyde	123-11-5	4.03	3.985	−0.0448
13#	*p*-Dimethylaminobenzaldehyde	100-10-7	5.4	4.622	−0.778
14#	Malononitrile	109-77-3	2.55	1.783	−0.767
15#	Glycolonitrile	107-16-4	2.98	2.141	−0.839
16#	α-Hydroxyisobutyronitrile	75-86-5	3.61	3.834	0.227
17#	Allyl cyanide	109-75-1	2.06	1.507	−0.553
18#	Benzonitrile	100-47-0	3.48	3.456	−0.0237
19#	Benzyl cyanide	140-29-4	4.23	2.963	−1.267
20#	Acetonitrile	1975-5-8	0.75	2.023	1.273
21#	Acrylonitrile	107-13-1	1.51	1.467	−0.0414
22#	Succinonitrile	110-61-2	0.36	2.401	2.042
23#	Phthalonitrile	91-15-6	3.51	2.622	−0.888
24#	Lactonitrile	78-97-7	2.01	2.440	0.430
25#	Atrazine	1912-24-9	6.68	7.543	0863
26#	Prometryn	7287-19-6	8.07	6.457	−1.613
27#	Simetryn	1014-70-6	6.29	5.565	−0.725
28#	Prometone	1610-18-0	8.99	7.801	−1.182
29#	Simazine	122-34-9	5.43	6.892	1.462
30#	Metribuzin	21087-64-9	5.7	6.873	1.173
31#	Cyanazine	21725-46-2	6.61	6.631	0.0212
32#	Terbutryn	886-50-0	7.95	6.131	−1.819
33#	Terbutylazine	5915-41-3	6.93	8.225	1.295
34#	Ametryn	834-12-8	6.56	5.516	−1.044
35#	Diuron	330-54-1	7.72	6.065	−1.655
36#	Chlorotoluron	15545-48-9	8.4	5.898	−2.502
37#	Monolinuron	1746-81-2	7.33	6.384	−0.946
38#	Monuron	150-68-5	6.3	6.254	−0.0460
39#	Methabenzthiazuron	18691-97-9	7.02	6.022	−0.998
40#	Isoproturon	34123-59-6	7.18	6.554	−0.627
41#	Fenuron	101-42-8	7.33	6.665	−0.665
42#	Ethametsulfuron	111353-84-5	4.13	6.302	2.172
43#	Chlorsulfuron	64902-72-3	6.42	6.337	−0.0833
44#	Metsulfuron	79510-48-8	6.29	6.245	−0.0447
45#	Sulfamethazine	57-68-1	4.08	5.506	1.426
46#	Sulfapyridine	144-83-2	3.84	3.407	−0.433
47#	Sulfamethoxazole	723-46-6	4.45	4.511	0.0609
48#	Sulfadiazine	68-35-9	4.5	5.021	0.521
49#	Sulfisoxazole	127-69-5	4.43	5.506	1.076
50#	Sulfamonomethoxine	1220-83-3	5.05	4.535	−0.515
51#	Sulfachloropyridazine	80-32-0	4.78	5.117	0.337
52#	Sulfachinoxalin	59-40-5	4.53	5.203	0.673
53#	Sulfamethoxydiazine	651-06-9	4.41	5.050	0.640
54#	Sulfamethoxypyridazine	80-35-3	4.36	4.934	0.579
55#	Trimethoprim	738-70-5	3.22	5.209	1.989

**Table 2:** Descriptors, Coefficients, Standard Error, and *t*-Test Values for the Best Multiple Linear Regression (MLR) Model.

Coefficients	Standard Error	*t*-Test	Descriptors	VIF	MF
−0.405	0.463	−0.874	Intercept		
−0.688	0.322	−2.137	Number of triple bonds (NTB)	2.210	−0.051
1.847	0.255	7.257	Average Complementary Information content (order 2) (ACIC2)	1.109	0.401
63.611	7.542	8.434	Max partial charge for a C atom (Zefirov’s PC) ($Q_{Max}^{C}$)	1.002	0.650

**Table 3:** Correlation matrix of the 3 descriptors used in the model.

	ACIC2	NTB	$Q_{Max}^{C}$
**ACIC2**	1.000		
**NTB**	−0.314	1.000	
$Q_{Max}^{C}$	0.740	0.013	1.000

**Table 4:** The statistical results of the external test set for the MLR and radial basis function neural network (RBFNN) models.

	MLR	RBFNN
R^2^	0.853	0.896
$q_{\mathrm{ext}}^{2}$	0.825	0.890
$R_{0}^{2}$	0.849	0.896
(R^2^−$R_{0}^{2}$)/R^2^	0.005	0.000
k	0.983	1.030
F	30.861	155.424
RMS	0.691	0.547

**Table 5:** The No., chemicals in the mixtures, ratio of toxic unit, experimental −log(EC_50mix_), predicted −log(EC_50mix_), and their corresponding residual.

No.	Chemicals in the Mixtures	The Ratio of Toxic Unit	Experimental−log(EC_50mix_) (mol/L)	MLR		RBFNN		
				Predicted−log(EC_50mix_) (mol/L)	Residual	Predicted−log(EC_50mix_) (mol/L)	Residual	Set*
1	1#:14#	1:1	2.44	2.36	−0.08	2.67	0.23	A
2	2#:14#	1:1	2.63	2.38	−0.25	2.68	0.05	B
3*	3#:14#	1:1	2.77	2.38	−0.39	2.69	−0.08	T
4	4#:14#	1:1	2.78	2.61	−0.17	2.77	−0.01	C
5	5#:14#	1:1	2.8	3.14	0.34	2.79	−0.01	D
6	6#:14#	1:1	2.84	2.61	−0.23	2.80	−0.04	A
7	7#:14#	1:1	2.84	3.33	0.49	2.70	−0.14	B
8*	8#:14#	1:1	2.83	2.75	−0.08	2.82	−0.01	T
9	9#:14#	1:1	2.84	2.75	−0.09	2.81	−0.03	C
10	10#:14#	1:1	2.85	2.70	−0.15	2.81	−0.04	D
11	11#:14#	1:1	2.83	2.84	0.01	2.82	−0.01	A
12	12#:14#	1:1	2.84	2.82	−0.02	2.82	−0.02	B
13*	13#:14#	1:1	2.85	3.18	0.33	2.78	−0.07	T
14	5#:15#	1:1	3.15	3.25	0.10	3.03	−0.12	C
15	6#:15#	1:1	3.26	2.72	−0.54	3.21	−0.05	D
16	7#:15#	1:1	3.25	3.44	0.19	3.17	−0.08	A
17	8#:15#	1:1	3.24	2.86	−0.38	3.11	−0.13	B
18*	9#:15#	1:1	3.26	2.85	−0.41	3.10	−0.16	T
19	10#:15#	1:1	3.27	2.80	−0.47	3.14	−0.13	C
20	11#:15#	1:1	3.22	2.95	−0.27	3.02	−0.20	D
21	13#:15#	1:1	3.28	3.29	0.01	3.06	−0.22	A
22	1#:16#	1:1	2.64	3.43	0.79	3.38	0.74	B
23*	2#:16#	1:1	2.97	3.45	0.48	3.38	0.41	T
24	3#:16#	1:1	3.39	3.46	0.07	3.38	−0.01	C
25	5#:16#	1:1	3.51	4.22	0.71	3.71	0.20	D
26	6#:16#	1:1	3.83	3.70	−0.13	3.42	−0.41	A
27	7#:16#	1:1	3.78	4.41	0.63	3.52	−0.26	B
28*	8#:16#	1:1	3.75	3.83	0.08	3.56	−0.19	T
29	9#:16#	1:1	3.83	3.82	−0.01	3.56	−0.27	C
30	10#:16#	1:1	3.86	3.78	−0.08	3.51	−0.35	D
31	11#:16#	1:1	3.7	3.92	0.22	3.67	−0.03	A
32	12#:16#	1:1	3.77	3.90	0.13	3.61	−0.16	B
33*	13#:16#	1:1	3.9	4.26	0.36	3.68	−0.22	T
34	1#:17#	1:1	2.18	2.10	−0.08	1.81	−0.37	C
35	3#:17#	1:1	2.33	2.13	−0.20	1.87	−0.46	D
36	4#:17#	1:1	2.34	2.35	0.01	1.93	−0.41	A
37	5#:17#	1:1	2.34	2.89	0.55	2.68	0.34	B
38*	6#:17#	1:1	2.36	2.36	0.00	2.28	−0.08	T
39	7#:17#	1:1	2.36	3.07	0.71	3.06	0.70	C
40	8#:17#	1:1	2.36	2.50	0.14	2.27	−0.09	D
41	10#:17#	1:1	2.36	2.44	0.08	2.25	−0.11	A
42	11#:17#	1:1	2.35	2.59	0.24	2.27	−0.08	B
43*	12#:17#	1:1	2.35	2.56	0.21	2.28	−0.07	T
44	13#:17#	1:1	2.36	2.93	0.57	2.74	0.38	C
45	5#:18#	1:1	3.45	4.01	0.56	4.06	0.61	D
46	6#:18#	1:1	3.72	3.48	−0.24	3.53	−0.19	A
47	7#:18#	1:1	3.68	4.19	0.51	3.88	0.20	B
48*	8#:18#	1:1	3.66	3.62	−0.04	3.76	0.10	T
49	10#:18#	1:1	3.74	3.56	−0.18	3.68	−0.06	C
50	11#:18#	1:1	3.62	3.71	0.09	3.94	0.32	D
51	12#:18#	1:1	3.67	3.68	0.01	3.86	0.19	A
52	13#:18#	1:1	3.78	4.05	0.27	4.03	0.25	B
53*	5#:19#	1:1	3.67	3.72	0.05	4.23	0.56	T
54	6#:19#	1:1	4.25	3.20	−1.05	3.25	−1.00	C
55	7#:19#	1:1	4.14	3.91	−0.23	4.23	0.09	D
56	8#:19#	1:1	4.08	3.34	−0.74	3.56	−0.52	A
67	9#:19#	1:1	4.26	3.33	−0.93	3.56	−0.70	B
58*	10#19#	1:1	4.36	3.28	−1.08	3.45	−0.91	T
59	13#:19#	1:1	4.5	3.76	−0.74	4.23	−0.27	C
60	25#:35#	1:1	6.94	7.10	0.16	7.78	0.84	D
61	25#:36#	1:1	6.97	7.01	0.04	7.63	0.66	A
62	25#:37#	1:1	6.89	7.28	0.39	6.95	0.06	B
63*	25#:38#	1:1	6.45	7.21	0.76	7.92	1.47	T
64	26#:35#	1:1	7.86	6.48	−1.38	6.76	−1.10	C
65	26#:36#	1:1	8.2	6.39	−1.81	6.90	−1.30	D
66	26#:37#	1:1	7.56	6.67	−0.89	7.64	0.08	A
67	26#:38#	1:1	6.59	6.60	0.01	6.45	−0.14	B
68*	27#:35#	1:1	6.58	5.97	−0.61	6.95	0.37	T
69	27#:36#	1:1	6.59	5.88	−0.71	6.81	0.22	C
70	27#:37#	1:1	6.55	6.16	−0.39	7.10	0.55	D
71	27#:38#	1:1	6.29	6.08	−0.21	7.03	0.74	A
72	28#:35#	1:1	8	7.25	−0.75	7.64	−0.36	B
73*	28#:36#	1:1	8.6	7.15	−1.45	7.79	−0.81	T
74	28#:37#	1:1	7.62	7.43	−0.19	7.90	0.28	C
75	28#:38#	1:1	6.6	7.36	0.76	7.31	0.71	D
76	29#:35#	1:1	5.73	6.73	1.00	6.12	0.39	A
77	29#:36#	1:1	5.73	6.64	0.91	6.38	0.65	B
78*	29#:37#	1:1	5.73	6.92	1.19	7.29	1.56	T
79	29#:38#	1:1	5.68	6.85	1.17	5.67	−0.01	C
80	45#:55#	1:1	5.08	5.45	0.37	5.86	0.78	D
81	46#:55#	1:1	4.85	4.26	−0.59	3.98	−0.87	A
82	47#:55#	1:1	5.5	4.89	−0.61	5.02	−0.48	B
83*	48#:55#	1:1	5.42	5.17	−0.25	5.56	0.14	T
84	49#:55#	1:1	5.45	5.45	0.00	6.06	0.61	C
85	50#:55#	1:1	6.01	4.90	−1.11	5.05	−0.96	D
86	51#:55#	1:1	5.73	5.23	−0.50	5.56	−0.17	A
87	47#:55#	13396:1	3.49	4.48	0.99	3.84	0.35	B
88*	47#:55#	8587:1	3.49	4.48	0.99	3.84	0.35	T
89	47#:55#	2747:1	3.49	4.48	0.99	3.84	0.35	C
90	47#:55#	858:1	3.51	4.48	0.97	3.84	0.33	D
91	47#:55#	274:1	3.55	4.49	0.94	3.85	0.30	A
92	47#:55#	85:1	3.67	4.49	0.82	3.86	0.19	B
93*	47#:55#	27:1	3.92	4.51	0.59	3.92	0.00	T
94	47#:55#	15:1	4.08	4.53	0.45	3.98	−0.10	C
95	47#:55#	4:1	4.52	4.64	0.12	4.32	−0.20	D
96	47#:55#	1:6	5.34	4.59	−0.75	4.18	−1.16	A
97	47#:55#	1:21	5.43	5.25	−0.18	5.37	−0.06	B
98*	47#:55#	1:37	5.45	5.27	−0.18	5.36	−0.09	T
99	47#:55#	1:116	5.46	5.28	−0.18	5.34	−0.12	C

*Test set. Set* “T” means the corresponding compound belongs to the external test set. Set*s “A”, “B”, “C”, and “D” mean the compound belongs to the subset of the training set.

**Table 6:** The R^2^ and LOOq^2^ values of 10 Y-randomization tests.

No.	MLR		RBFNN	
	R^2^	LOOq^2^	R^2^	LOOq^2^
1	0.028	0.016	0.019	0.006
2	0.013	0.000	0.027	0.014
3	0.02	0.007	0.017	0.004
4	0.047	0.035	0.035	0.022
5	0.03	0.017	0.034	0.022
6	0.014	0.002	0.024	0.011
7	0.034	0.022	0.017	0.004
8	0.013	0.001	0.04	0.028
9	0.018	0.005	0.024	0.012
10	0.049	0.036	0.014	0.001
Average	0.0266	0.0141	0.0251	0.0124

**Table 7:** Validation of the MLR model.

Training Set	R^2^	F	RMS	Test Set	R^2^	F	RMS
A+B+C+D	0.869	165.290	0.599	T	0.853	30.861	0.691
B+C+D+T	0.857	150.314	0.633	A	0.904	50.357	0.527
A+C+D+T	0.864	159.122	0.611	B	0.883	40.100	0.611
A+B+D+T	0.867	162.819	0.600	C	0.866	32.528	0.674
A+B+C+T	0.868	167.263	0.594	D	0.856	29.716	0.713
Average	0.865	160.962	0.607		0.872	36.712	0.643

**Table 8:** Validation of the RBFNN model.

Training Set	R^2^	F	RMS	Test Set	R^2^	F	RMS
A+B+C+D	0.925	950.686	0.447	T	0.896	155.424	0.547
B+C+D+T	0.915	827.525	0.494	A	0.932	247.421	0.418
A+C+D+T	0.910	778.942	0.519	B	0.954	371.815	0.361
A+B+D+T	0.915	824.679	0.500	C	0.931	244.294	0.455
A+B+C+T	0.921	911.329	0.472	D	0.900	152.589	0.559
Average	0.917	858.632	0.486		0.923	234.309	0.468
